# Real world clinicopathologic observations of patients with metastatic solid tumors receiving immune checkpoint inhibitor therapy: Analysis from Kentucky Cancer Registry

**DOI:** 10.1002/cam4.3802

**Published:** 2021-02-22

**Authors:** Aasems Jacob, Jianrong Wu, Jill Kolesar, Eric Durbin, Aju Mathew, Susanne Arnold, Aman Chauhan

**Affiliations:** ^1^ Department of Internal Medicine University of Kentucky Lexington KY USA; ^2^ Department of Biostatistics University of Kentucky Lexington KY USA; ^3^ College of Pharmacy University of Kentucky Lexington KY USA; ^4^ Cancer Research Informatics Shared Resource Facility Markey Cancer Center University of Kentucky Lexington KY USA

**Keywords:** biomarkers, immune checkpoint inhibitors, immunotherapy, immunotherapy response, solid tumors

## Abstract

The state of Kentucky has the highest cancer incidence and mortality in the United States. High‐risk populations such as this are often underrepresented in clinical trials. The study aims to do a comprehensive analysis of molecular landscape of metastatic cancers among these patients with detailed evaluation of factors affecting response and outcomes to immune checkpoint inhibitor (ICI) therapy. We performed a retrospective analysis of metastatic solid tumor patients who received ICI and underwent molecular profiling at our institution.

Sixty nine patients with metastatic solid tumors who received ICI were included in the study. Prevalence of smoking and secondhand tobacco exposure was 78.3% and 14.5%, respectively. TP53 (62.3%), CDKN1B/2A (40.5%), NOTCH and PIK3 (33.3%) were the most common alterations in tumors. 67.4% were PDL1 positive and 59.4% had intermediate‐high tumor mutational burden (TMB). Median TMB (12.6) was twofold to fourfold compared to clinical trials. The prevalence of mutations associated with smoking, homologous recombinant repair and PIK3/AKT/mTOR pathway mutations was higher compared to historic cohorts.

PDL1 expression had no significant effect on radiologic response, but PFS improvement in patients with tumors expressing PDL1 trended toward statistical significance (median 18 vs. 40 weeks. HR = 1.43. 95%CI 0.93, 4.46). Median PFS was higher in the high‐TMB cohort compared to low‐intermediate TMB (median not reached vs. 26 weeks; HR = 0.37. 95%CI 0.13, 1.05). A statistically significant improvement in PFS was observed in the PIK3 mutated cohort (median 123 vs. 23 weeks. HR = 2.51. 95%CI 1.23, 5.14). This was independent of tumor mutational burden (TMB) status or PDL1 expression status. PIK3 mutants had a higher overall response rate than the wild type (69.6% vs. 43.5%, OR 0.34; *p* = 0.045). The results should prompt further evaluation of these potential biomarkers and more widespread real‐world data publications which might help determine biomarkers that could benefit specific populations.

## Graphical abstract

Retrospective analysis of tumor mutational landscape assessment and its impact on immunotherapy response in metastatic solid tumor patients with high‐smoking prevalence.

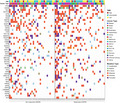

## 1 BACKGROUND

Kentucky has the highest cancer incidence and mortality rates in the country with mortality rates nearly 50% higher than the national average.^1^ Although this has been mainly attributed to the high‐smoking prevalence in the state, economic, and health care disparities, and genetic factors also play a key role. High‐risk populations such as this are often underrepresented in clinical trials. This could be the reason behind variation of smoking status, predictive biomarkers and tumor genomic alteration between real‐world and clinical trials.^2^

Immune checkpoint inhibitors (ICI) were developed to counter the upregulation of immune checkpoints by tumors by targeting programmed cell death protein (PD‐1)/programmed death‐ligand 1 (PDL‐1) or common T lymphocyte antigen‐4 (CTLA‐4), resulting in recognition and killing of tumor cells by the host immune system. There are over two dozen FDA‐approved indications for this class of drugs currently, and they are being increasingly studied in various clinical trials, gradually broadening indications. With accumulating data, it has become clear that the treatment benefits are not homogenous among all the patients. Response rates to single‐agent PD‐1/PDL‐1 inhibitors are variable with 40% in melanoma, 25% in non‐small cell lung cancer (NSCLC), and 19% in renal cell carcinoma.^3^, ^4^, ^5^, ^6^, ^7^ This highlights the importance of finding a discriminating biomarker that could predict the outcomes of ICI therapy. PDL1 expression, microsatellite instability status, tumor mutation burden, CD8+ T cell infiltration are some of the extensively studied predictive biomarkers; however, with conflicting data in different studies.^8^, ^9^ The variation in patient selection criteria, assays and determinants used to assess PDL1 expression could have contributed to this discrepancy.^10^ Immune‐related adverse events (iRAEs) from ICI therapy affect patients differently, and it is critical to understand its determinants. Evaluation of the current conflicting data on the association between the incidence of iRAEs and response to ICI is equally essential.^11^, ^12^ Most importantly, there is a lack of real‐world data on response to ICI response and patient outcomes in diverse populations. Real‐world datasets, which reveal genomic profiling and clinical outcomes, provide an important tool to recognize inferences on biomarkers of response and resistance. These data could also enable the discovery of novel biomarkers not be seen in the highly selective clinical trials. Randomized controlled trials with strict entry requirements to guarantee internal stability can lead to the loss of external scalability. This could be due to the exclusion of patients with poor prognosis, older patients, patients with brain metastases, and an ECOG score of 2 or more. Mutational analyses have now enabled subclassification of tumors into molecularly defined subtypes. Evaluation of ICI response and resistance in tumors that demonstrate these mutations is a muddy landscape with little valuable data.

Our study aims to assess various molecular markers and their impact on progression‐free survival and radiological response rates with ICI therapy. This assessment is especially important in our population as Kentucky has an exceedingly high‐smoking prevalence of 24.6% among adults and has the highest incidence of cancer in the United States. This population could present a varied biomarker profile and responses compared to that seen in clinical trials and provide insight into new biomarkers. In recent studies, a higher tumor mutational burden was identified among patients who smoke, with the possibility of smoking‐specific mutations as drivers for oncogenesis.^13^ Although the results might not be generalizable, they can give potential information to further search for biomarkers and help identify specific signatures in this high‐risk population.

## 2 METHODOLOGY

We conducted a retrospective analysis of all patients with metastatic solid tumors above 18 years of age who received ICI in our institution between January 2016 and January 2020. The cancer center is the highest volume cancer center and the only National Cancer Institute designated cancer center in the state of Kentucky. The institutional review board approved the study. Demographics, treatment plans, and outcomes were obtained by reviewing electronic health records and Kentucky Cancer Registry (KCR). US Food and Drug administration approved next‐generation sequencing (NGS) platforms‐ FoundationOne^®^ CDx and FoundationOne^®^ Dx panels were used for mutational analysis of tumor samples. These tests can be used to detect substitutions, insertions, deletions, and copy number alterations in 324 genes and selected gene rearrangements. The system also estimates tumor mutational burden and microsatellite instability (MSI) using DNA isolated from tumor tissue specimens. MSI status is determined by a genome‐wide analysis of 95 microsatellite loci. PDL1 expression is tested using PD‐L1 22C3 IHC with interpretation using Tumor Proportion Score (TPS). Tumor mutational burden (TMB) is reported as mutations per megabase (mut/Mb) unit. It is further subdivided into high (≥20Muts/Mb), intermediate (6–19 Muts/Mb), and low (≤5Muts/Mb). Patients with indeterminate TMB were excluded from the analysis. For analysis, TMB was dichotomized as high and low to intermediate. Only genomic alterations of known significance were included in the analysis. One investigator assessed radiologic response to ICI therapy for all patients using Response Evaluation Criteria for Solid Tumors (RECIST) 1.1 by analyzing radiology reports and image records. Response categories were divided into complete response (CR), partial response (PR), stable disease (SD), and progression of disease (PD). 146 patients who did not have complete molecular and radiological information or lost to follow up before complete evaluation were excluded from the analysis. Progression‐free survival (PFS), radiological response, and autoimmune side effects were analyzed with various molecular biomarkers.

### 2.1 Statistical analysis

Categorical variables were analyzed as percentages and continuous variables as means or medians. Statistical analysis was performed using SPSS 26.0 and JMP 14 software. Logistic regression, Fisher's exact test, Kaplan‐Meier method, log‐rank test, and Cox regression were used to analyze clinical features and efficacy outcomes. *p*‐value <0.05 was considered to be statistically significant. Overall survival was not considered for analysis as there were only eight deaths, probably due to a shorter period of follow‐up.

## 3 RESULTS

### 3.1 Patient characteristics

Sixty nine patients had genomic and radiologic data for inclusion in the study. The median follow‐up period was 63 weeks. 36 (52.2%) subjects were male. The mean age of the study population was 62 (range: 27–95 years), and 14.5% were over 75 years of age. 91.3% were of Caucasian ethnicity. There was a very high prevalence of smoking (78.3%), and 66.7% of the never‐smokers also had recorded secondhand smoke exposure. 13% of the study population also used e‐cigarettes. 100% of patients with NSCLC were smokers. 45% of patients were from Appalachian region and 60% from rural counties. NSCLC constituted the majority (37.7%) of the tumor types included in the study, followed by squamous cell cancer (SCC) of the head and neck and melanoma. 23% of the patients had brain metastasis at diagnosis or on progression. 100% of patients with NSCLC were smokers (Table 1).

**TABLE 1:** Baseline characteristics of the study population

Characteristics	Total (%)
Age group	
18–64	40 (58)
65–74	19 (27.5)
≥ 75	10 (14.5)
Male: Female	36 (52.2): 33 (47.8)
Race	
Caucasian	63 (91.3)
African American	4 (5.8)
Hispanic	1 (1.4)
Asian	1 (1.4)
Cancer Site	
NSCLC	26 (37.7)
SCC Head & Neck	8 (11.6)
Melanoma	7 (10.1)
Gastrointestinal	7 (10.1)
Kidney/Bladder	6 (8.6)
Small Cell lung Cancer	5 (7.2)
Hepatobiliary	2 (2.9)
Breast	2 (2.9)
Ovary and uterus	2 (2.9)
Thyroid	2 (2.9)
Merkel Cell	1 (1.4)
Unknown primary	1 (1.4)
Tobacco use	
Current/previous use	54 (78.3)
Never used	15 (21.7)
Secondhand exposure	10 (14.5)

Abbreviations: NSCLC, Non‐small cell lung cancer; SCC, Squamous cell carcinoma.

### 3.2 Treatment

Mean time to ICI use from diagnosis was 69.5 weeks. Pembrolizumab was the most commonly used ICI (65%) followed by nivolumab and ipilimumab (16% each), and most patients received single‐agent immunotherapy (77%). 40 patients (58%) received ICI treatment as first line therapy, 23 (33.3%) as second line and 6 (8.9%) received as third line for FDA‐approved indications. None of the patients received ICI for high‐TMB or MSI‐H status. About half of these patients (47.8%) had iRAEs with grade 3/4 adverse events reported at 14.4%. Hyperprogression, which is treatment failure within two months of treatment initiation or ≥50% increase in tumor burden in 2 diameters or ≥100% in one diameter, was present in six patients (8.7%). Radiologic pseudo‐progression was found only in 2 patients, one with NSCLC and one with squamous cell cancer of head and neck (Table 2).

**TABLE 2:** Treatment and biomarker characteristics of study population

Characteristics	*n* (%)
ICI	
Atezolizumab	2 (2.9)
Ipilimumab‐Nivolumab	11 (15.9)
Nivolumab	11 (15.9)
Pembrolizumab	45 (65.2)
IRAEs	
Any	33 (47.8)
Grade 1	7 (10.1)
Grade 2	16 (23.2)
Grade 3/4	10 (14.4)
Tumor Mutational Burden	
≥20 mut/Mb	9 (13)
6–19 mut/Mb	32 (46.4)
≤ 5 mut/Mb	28 (40.6)
PDL1 tumor proportion score	
0	18 (26.1)
1–49%	28 (40.6)
≥50	17 (24.6)
Not able to assess	6 (8.7)
Microsatellite stability	
Stable	61 (88.4)
Unstable	6 (8.7)
Unknown	2 (2.9)
Best Response	
CR	14 (20.3)
PR	22 (31.9)
SD	12 (17.4)
PD	21 (30.4)

### 3.3 Biomarkers and Mutational assay

**FIGURE 1:**
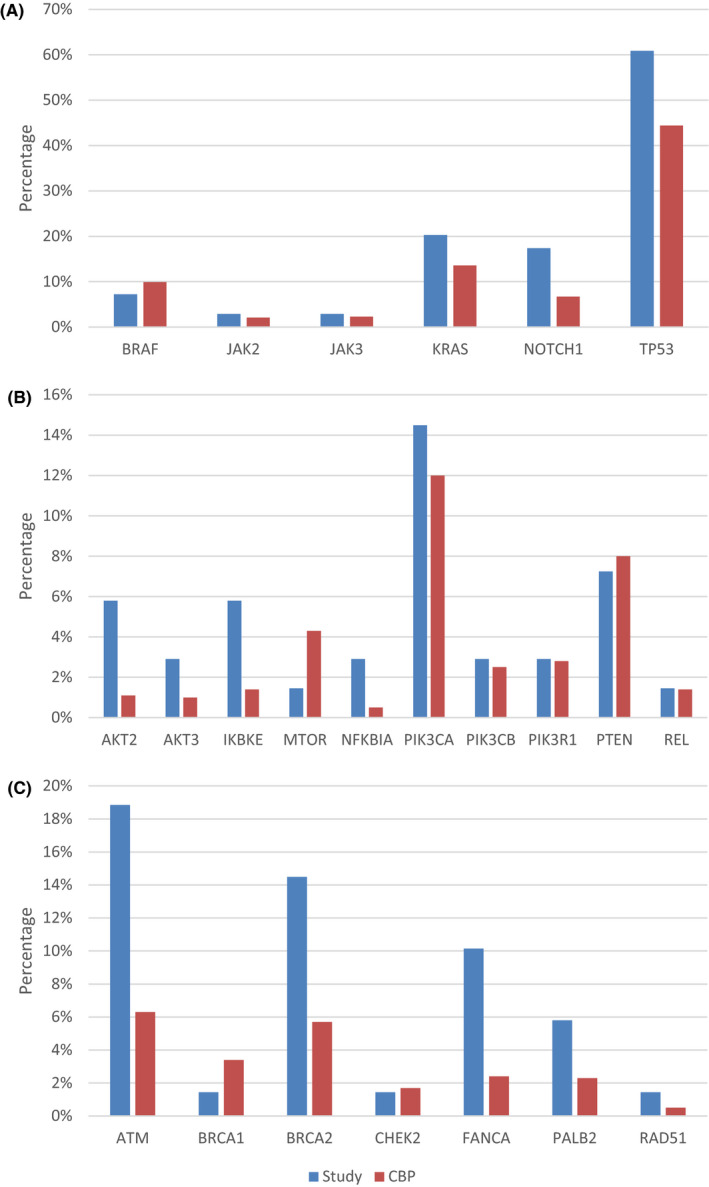
Percentage of patients with mutations in cBioPortal and this study. (A) NSCLC cohort in both studies with genomic alterations associated with smoking. (B) Genomic alterations involving PIK3/AKT/mTOR pathway. (C) Alterations implicated in homologous recombinant repair. (CBP: cBioPortal)

**FIGURE 2:**
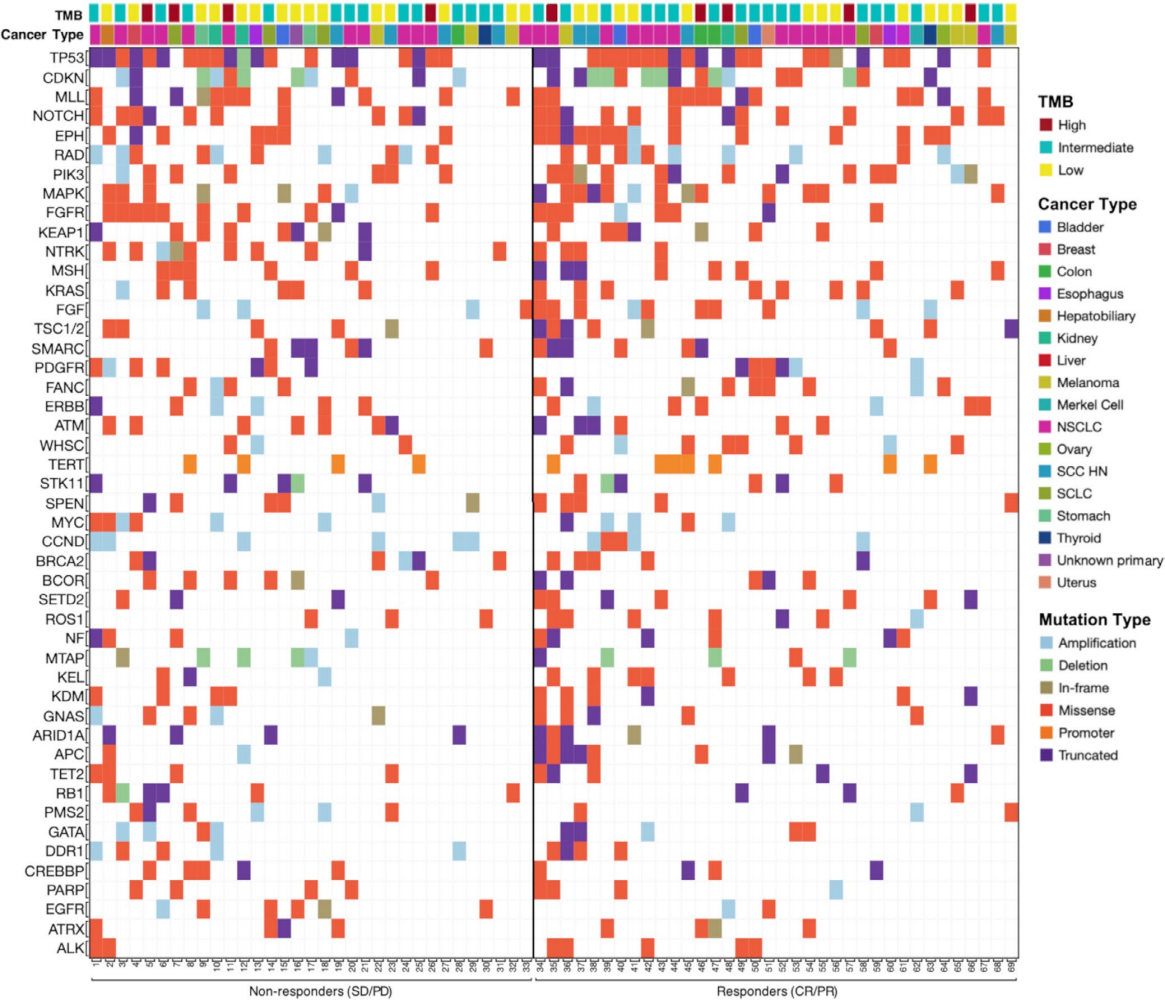
Mutational analysis of patients receiving immunotherapy grouped based on radiologic response, in the order of mutational load and frequency of mutations

The majority of the tumor samples (65.2%) had a positive PDL‐1 expression represented as a tumor proportion score (TPS) ≥1%. 90% of the tumors were microsatellite stable, and the average TMB was 12.6 (range 0–117). 13% of patients had TMB ≥20 mut/Mb and 46.4% tumors had TMB between 6–19 mut/Mb. The NGS panel detected TP53 as the most common (62.3%) tumor mutation. It was followed by CDKN1B/2A (40.5%), NOTCH (33.3%), and PIK3CA/2B (33.3%) mutations. STK11 mutation was found in 16% of patients. TMB did not correlate to smoking status or pack years (Table 2, Figure 1, Figure 2).

Tumor mutations commonly associated with smoking (BRAF, JAK2, JAK3, NOTCH1, TP53) were higher in the study population compared to cBioPortal database metastatic solid tumor cohorts.^14^, ^15^ There was similar difference on evaluation of genomic alterations involving PIK3/AKT/mTOR pathway and alterations implicated in homologous recombinant repair. BRCA1, MTOR, and PTEN mutations were, however, less common in the study group compared to the trial cohort.

### 3.4 Response and outcome by PDL1 expression and TMB

**FIGURE 3:**
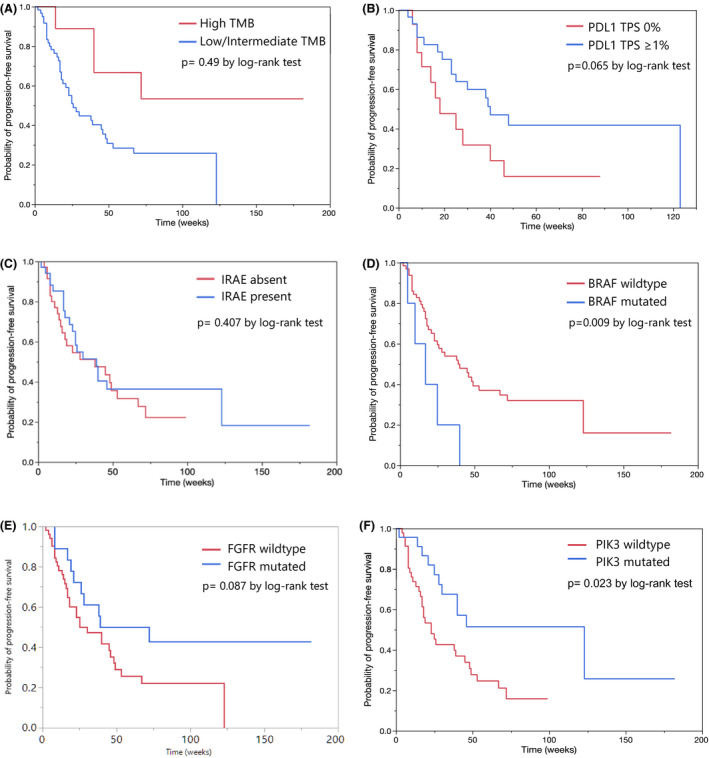
Kaplan‐Meier graphs depicting progression‐free survival in patients based on tumor samples showing (A) High TMB and low/intermediate TMB; (B) PDL1 expression; (C) Presence of IRAEs; (D) Presence of PIK3 mutation; (E) Presence of FGFR mutation; (F) Presence of BRAF mutation

TMB was not associated with smoking status or pack years. Patients with high TMB (≥20 mut/Mb) had an overall radiologic response rate (CR+PR) of 55.6% compared to 51.7% in low‐intermediate TMB group (*p* = 0.83). Median PFS was higher in high TMB compared to the low‐intermediate group and the results reached statistical significance (median not reached vs. 26 weeks; HR=0.37. 95%CI 0.13, 1.05). Although there was improvement in PFS (median 53 vs. 26 weeks, HR=1, 95%CI 0.37, 2.72) this was not statistically significant when the FDA‐approved cut off of ≥10mut/Mb for ICI use was used to identify high TMB. Radiologic responders were identical in both groups. (0.52% vs. 0.52%, OR = 1, *p* = 1). PDL1 expression was not associated with radiologic response, but there was a trend toward improved PFS in patients with tumors expressing PDL1 (median 18 vs. 40 weeks. HR = 1.43. 95%CI 0.93, 4.46). There was no significant correlation between size change of the target lesion with ICI therapy and absolute TMB values (R^2^ = 0.023) (Figure 3, Table 3).

**TABLE 3:** Table showing ORR based on various factors with odds ratio calculated using logistic regression model

Variable	Total	Responders (CR/PR) *n* (%)	Non‐responders (SD/PD) *n* (%)	Overall response rate	OR 95% CI
All patients	69	36 (52.2)	33 (47.8)	52.2%	
Age <65	40	19 (47.5)	21 (52.5)	47.5%	0.64 (0.24–1.68)
Age >65	29	17 (58.6)	12 (41.4)	58.6%	
Male	36	16 (44.4)	20 (55.6)	44.4%	1.92 (0.74–5.02)
Female	33	20 (60.6)	13 (39.4)	60.6%	
Smoker	54	26 (48.1)	28 (51.9)	48.1%	2.15 (0.64–7.14)
Non‐smoker	15	10 (66.7)	5 (33.1)	66.7%	
IRAE present	34	19 (55.9)	15 (44.1)	55.9%	0.75 (0.29–1.92)
IRAE absent	35	17 (48.6)	18 (51.4)	48.6%	
High TMB	9	5 (55.6)	4 (44.4)	55.6%	1.17 (0.29–4.78)
Low/int TMB	60	31 (51.7)	29 (48.3)	51.7%	
TMB <10	46	24 (52.2)	22 (47.8)	52.2%	1.00 (0.36–2.72)
TMB ≥10	23	12 (52.2)	11 (47.8)	52.2%	
PDL1 0%	18	6 (33.3)	12 (64.7)	33.3%	0.45 (0.12–1.68)
PDL1 >1%	45	25 (55.6)	20 (44.4)	55.6%	
PIK3 mutated	23	16 (69.6)	7 (30.4)	69.6%	**0.34 (0.12**–**0.97)**
PIK3 wild type	46	20 (43.5)	26 (56.5)	43.5%	
FGFR mutated	16	13 (72.2)	5 (27.8)	72.2%	0.32 (0.98–1.01)
FGFR wild type	51	23 (45.1)	28 (54.9)	45.1%	
ROS1 mutated	10	9 (90.0)	1 (10.0)	90.0%	**0.09 (0.01**–**0.79)**
ROS1 wild type	59	27 (45.8)	32 (54.2)	45.8%	
BRAF mutated	5	3 (60.0)	2 (40.0)	60.0%	0.71 (0.11–4.54)
BRAF wild type	64	33 (51.6)	31 (48.4)	51.6%	
STK 11 mutation	11	4 (36.4)	7 (63.6)	36.4%	2.15 (0.57–8.17)
STK11 wild type	58	32 (55.2)	26 (44.8)	55.2%	

Bold indicates statistically significant values.

### 3.5 Response and outcome by tumor mutations

The presence of BRAF mutation conferred shorter PFS with immunotherapy (median 17 vs. 39 weeks. HR = 0.35. 95%CI 0.14, 0.91) but had no significant association with radiologic response. STK11 mutation did not have a significant impact on PFS (median 30 vs. 39 weeks, HR=0.88. 95%CI 0.39, 2.0) or radiologic response (OR = 1.61, *p* = 0.58). Presence of KRAS mutations also did not show significant impact on PFS (median 48 vs. 38 weeks, HR=1.27, 95%CI 0.62, 2.58) or radiologic response (OR = 1.12, *p* = 0.84) (Figure 3, Table 3).

Statistically significant improvement in PFS was observed in the PIK3 mutated (PIK3CA/PIK3C2B) group (median 123 vs. 23 weeks. HR = 2.51. 95%CI 1.23, 5.14). (Figure 3) This was independent of TMB status or PDL1 expression status (HR 3.24, *p* = 0.016). Patients with PIK3 mutations had a higher overall response rate (ORR) than the unmutated group (69.6% vs. 43.5%, OR 0.34; *p* = 0.045). PIK3 mutated patients also had a higher risk of developing IRAEs (73.9% vs. 37%, OR = 0.21 *p* = 0.005), but the presence of mutation did not associate with TMB, PDL1 expression or microsatellite stability status ruling out collinearity.

There was a difference in ORR with FGFR mutations (72.2% vs 45.1%, OR 0.32; *p* = 0.05) and ROS1 mutation (90% vs. 45.8%, OR 0.09; *p* = 0.029). With FGFR mutation, there was a trend toward statistical significance for PFS with immunotherapy (median 39 vs. 30 weeks. HR = 1.84. 95%CI 0.90, 3.76), which was not seen with ROS1 mutation.

### 3.6 Response and outcome (others)

There was no statistically significant difference in radiologic response and PFS with ICI therapy based on smoking status, ICI drug, MSS status, incidence of IRAEs, or age (below and above 65 years of age).

## 4 DISCUSSION

Our study was aimed at evaluating clinicopathologic characteristics and outcomes in a subset of population with the highest incidence of cancer in the country and often underrepresented in clinical trials. The outcomes with immunotherapy, incidence of iRAEs and biomarker profiling of these patients are poorly understood. With the current data available only from a carefully selected clinical trial population, many biomarkers that could be effective in such selected population might have been overlooked. The study also looked at potential markers of immunotherapy response in this patient population (90).

The prevalence of smoking and secondhand smoke exposure was high in the study group. The study did not find association of smoking status with TMB. Nearly half (47.8%) of the patients experienced any degree of IRAEs. The median tumor mutation burden was 12.6 mutations/Mb which was at least twofold to fourfold compared to other similar studies.^16^, ^17^ Our results show the prevalence of PDL1 expression, TMB in our study group are similar to ones seen in previous clinical trials. Compared to historic cohorts, the prevalence of PIK3/AKT/mTOR pathway and homologous recombinant repair alterations were higher in the study group. Smoking associated tumor genomic alterations was also significantly higher compared to similar cohorts.

High‐tumor mutational burden, and the presence of PIK3 mutation conferred better progression‐free survival with immunotherapy across cancer types. This PFS benefit seen in PIK3 mutated patients was independent of PDL1 status or TMB. The effect of PDL1 expression and FGFR mutation on PFS trended toward statistical significance. The presence of PIK3 mutation and ROS1 mutation also had a statistically significant favorable impact on the best overall radiologic tumor response while with FGFR mutation, this trended toward statistical significance. The high incidence of IRAEs in the PIK3 mutated group also points toward possible increased PDL1 inhibitor activity. STK11 mutation, which was previously implicated in immunotherapy resistance, did not significantly affect immunotherapy response or outcomes. Although the absolute numbers were small, patients with BRAF mutations had a poor outcome with immune therapy.

The PIK3/AKT/mTOR (PAM) pathway is considered a master regulator of cancer and plays a vital role in tumor growth, proliferation, angiogenesis, and cell survival.^18^ PIK3CA is the most studied among PIK3 mutations, with amplification or clustering of somatic mutations occurring in up to 30% of endometrial, breast, ovarian, and colon cancers while PIK3CB is found in thyroid and lung cancers.^19^ PI3KCA/CB mutations are primarily activating, while the pathway inhibiting PIK3R1 mutations are mostly inactivating.^20^ These mutations were also implicated in chemotherapy and HER2 blocker resistance.^21^, ^22^ The pathway, more importantly, plays a stimulatory role in PDL1 transcription and expression on tumor cells.^23^, ^24^ Despite the abundance of laboratory and animal data, clinical data linking various PIK3 protein mutations and immunotherapy responses are lacking.

The study has limitations with small sample size, short follow‐up period and being a single‐center study. Most of the patients who received ICI at the institution were not included in the final analysis due to incomplete data or loss to follow up making the need of prospective trials important. The results should prompt further evaluation of these potential biomarkers and more widespread real‐world data publications.

## 5 CONCLUSION

The study characterizes the ICI response, IRAEs, and mutational profile in a population with high prevalence of smoking and the highest cancer incidence and mortality in the country. The prevalence of mutations associated with smoking, homologous recombinant repair and PIK3/AKT/mTOR pathway were higher in the study group compared to similar cohorts. The previously reported positive evidence for TMB and PDL1 expression as ICI biomarkers were replicated in this study. The presence of PIK3 mutation conferred better progression‐free survival and radiologic response with immunotherapy across cancer types. Although conflicting evidence exists on using these markers across cancers for patient selection, these could still be relevant in specific target populations with significant risk factors like smoking in our population. More extensive studies and long‐term follow‐up data are needed to confirm the clinical validity and role of individual mutations in immunotherapy response. Furthermore, more real‐world data is needed to help identify specific biomarkers based on population and risk factors.

## CONFLICTS OF INTEREST

Authors Aasems Jacob, Jianrong Wu, Jill Kolesar, Eric Durbin, Aju Mathew and Susanne Arnold express no conflicts of interest. Aman Chauhan has received research grant from BMS, Clovis, Lexicon; Advisor Lexicon, Ipsen.

## ETHICAL APPROVAL AND INFORMED CONSENT

- University of Kentucky Institutional Review Board approved the study (IRB# #49450 on 8/9/2019)
- The Office of Research Integrity under IRB, University of Kentucky authorized waiver of informed consent as the study involved no more than minimal risks for the subjects and their privacy.

## Data Availability

Raw data were generated at University of Kentucky and Kentucky Cancer Registry. Derived data supporting the findings of this study are available from the corresponding author AJ on request.
